# Non-invasive *in vivo* fluorescence imaging of apoptotic retinal photoreceptors

**DOI:** 10.1038/s41598-018-38363-z

**Published:** 2019-02-07

**Authors:** Francesca Mazzoni, Claudia Müller, Jonathan DeAssis, Deborah Lew, W. Matthew Leevy, Silvia C. Finnemann

**Affiliations:** 1 Department of Biological Sciences, Center for Cancer, Genetic Diseases and Gene Regulation, Fordham University, Bronx, NY, 10458 USA; 2 Department of Biological Sciences, 100 Galvin Life Science Center, University of Notre Dame, Notre Dame, IN 46556 USA

## Abstract

Phosphatidylserine externalization is an early molecular signature for apoptosis. In many retinal degenerative diseases, photoreceptor neurons die by apoptosis. Here, we report utility of the phosphatidylserine-binding conjugate of Bis(zinc(II)-dipicolylamine (Zn-DPA) with Texas-red (PSVue-550) in transiently labeling apoptotic photoreceptors in living pigmented or albino rats and mice with retinal degeneration. Applying PSVue-550 as eyedrop is non-toxic and eliminates need for intraocular injection. PSVue-550 fluorescence specifically and transiently labeling dying retinal photoreceptors is detectable in anesthetized animals using standard retinal or whole small animal imaging systems. Importantly, prior PSVue-550 eyedrop administration and imaging does not affect repeat testing. Altogether, our results establish PSVue-550 imaging as a completely non-invasive method that provides the opportunity to longitudinally monitor retinal photoreceptor cell death in preclinical studies.

## Introduction

Apoptosis is a regulated form of programmed cell death that plays an essential role in numerous physiological processes and diseases including hereditary and induced forms of retinal degeneration^1,2^. During early apoptosis, enzymatic translocation of anionic phosphatidylserine (PS) from the inner to the outer leaflet of the plasma membrane serves as an “eat me” signal, which triggers clearance phagocytosis of apoptotic cells^3^. Detection of apoptosis in retinal degenerations is of critical importance in diagnosis, treatment, and monitoring of these debilitating diseases.

Bis(zinc(II)-dipicolylamine) (Zn-DPA) is a small (1.84 kDa) synthetic compound that binds to anionic phospholipids including PS. Zn-DPA conjugation to fluorophores yields probes (commercialized as PSVue®) that are suitable for PS live maging^4–6^. PSVue-480 (like annexin-V-protein probes^7^) administered by intravitreal injection successfully labels dying retinal ganglion cells, the innermost retinal neurons that directly neighbor the vitreous injection site^8^. Utility of non-invasive PS probes in labeling apoptotic photoreceptors, the outermost retinal neurons, has not been reported to date. Here, we show that Texas-red-conjugated PSVue (PSVue-550) detects photoreceptor apoptosis in living mice and rats when administered as an eyedrop. This procedure avoids intraocular injection, which may itself alter the retinal degenerative process.

## Results

### Specific PSVue-550 labeling of apoptotic photoreceptors 24 hours after application as eyedrop

**Figure 1:**
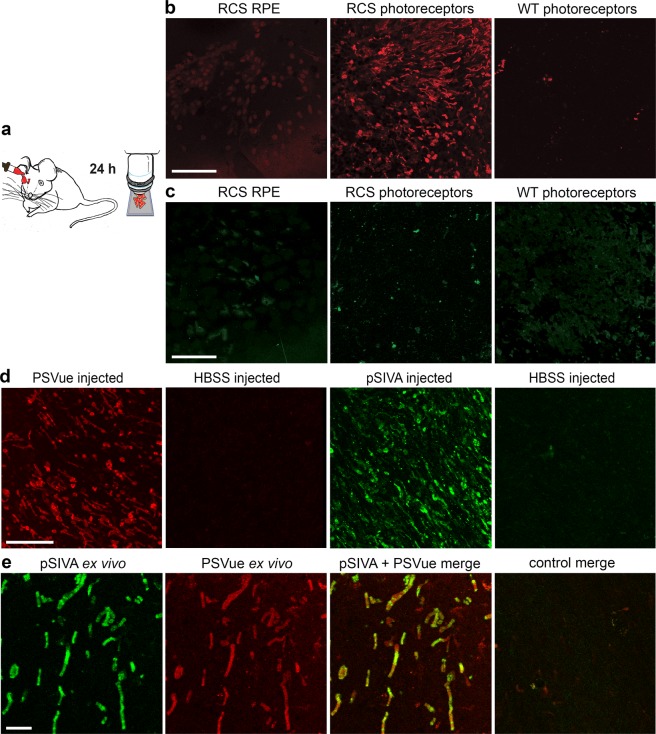
Comparison of staining of apoptotic photoreceptors by fluorescent PS probes PSVue-550 and pSIVA applied as eyedrop, by intravitreal injection, or to retina *ex vivo*. (**a**) Experimental paradigm for eyedrop probe application. (**b**,**c**) Representative maximal projection images of live dissected retina or RPE, as indicated, of PSVue-550 (**b**) or pSIVA (**c**) staining of p25 RCS or WT rat tissues as indicated. Tissues of equal volumes were imaged live immediately following dissection 24 hours after eye drop application as in **a**. Scale bar, 50 μm. (**d**) Representative images of live dissected retina after PSVue-550 or pSIVA intravitreal injection. Images from control eyes injected with HBSS buffer are also shown to the right of each fluorescent probe, as indicated. Scale bar, 20 μm. (**e**) Representative live imaging of co-staining by PSVue-550 and pSIVA probes following application of mixed probes to freshly dissected RCS rat retina *ex vivo*. Scale bar, 10 μm. All retina flatmounts are shown photoreceptor side up.

To test whether PSVue-550 has utility as *in vivo* apoptosis indicator, we first assessed eye penetration in a well characterized rat model of retinal degeneration, the Royal College of Surgeons (RCS) rat (RCS-rdy-p, pink-eyed)^9^. RCS rats lack photoreceptor outer segment renewal due to disruption of the *mertk* gene, which encodes a key clearance phagocytosis receptor. This results in rapid, synchronized photoreceptor death by apoptosis beginning around postnatal day 25 (p25)^9–11^. Indeed, P25 RCS rats showed intact retinal morphology with conserved inner and outer segments similar to age-matched wild-type (WT) rats (Supplementary Fig. S1). We thus explored p25 rats for PSVue-550 testing. We applied the probe as eyedrop to anesthetized RCS and WT rats. Rats were sacrificed 24 hours later, and neural retinas and posterior eyecups were dissected and immediately imaged live, mounted with either photoreceptors or retinal pigment epithelium (RPE) tissue side up (Fig. 1a). Fluorescence was only detected in the neural retina of RCS rats, indicating that PSVue-550 applied to the ocular surface reaches the photoreceptors and specifically labels apoptotic cells (Fig. 1b). To test if PSVue-550 penetrates the eye equally in WT and RCS rats, we quantified PSVue-550 in external rinse (to account for remaining free dye) before opening the eyeball and internal rinse (containing likely mostly vitreous) obtained from the posterior aspect of the eye following removal of the anterior segment 3 hours after eyedrop administration. ~4-fold higher PSVue-550 concentration inside as compared to outside the eye and similar levels of PSVue-550 in WT and RCS rat eyes (*P* = 0.621) showed that eyedrop-administered PSVue-550 penetrates the anterior tissues of the eye to reach the posterior eyeball irrespective of retinal degeneration (Supplementary Fig. S2). We also tested utility as eyedrop of a fluorescent PS biosensor that is an annexin derivative, “Polarity Sensitive Indicator of Viability & Apoptosis” (pSIVA)^12,13^. However, following pSIVA eyedrop application we did not detect specific pSIVA fluorescence of RCS apoptotic photoreceptors (Fig. 1c). In contrast, when either PSVue-550 or pSIVA were injected intravitreally, both dyes labeled apoptotic photoreceptors in RCS retina and exhibited similar staining patterns (Fig. 1d). Thus, lack of labeling following pSIVA administration as eyedrops was likely due to its inability to reach the outer retina. Finally, we co-stained freshly excised RCS rat retina with a mix of PSVue-550 and pSIVA followed by immediate live imaging as flat mount specimen. Both dyes labeled the same cells and structures including those with appearance of outer segments in these *ex vivo* experiments further supporting the staining specificity of PSVue-550 for apoptotic photoreceptors in the degenerating RCS retina (Fig. 1e).

### Lack of direct retinal toxicity of PSVue-550

To test toxicity of PSVue-550, we performed scotopic full-field electroretinograms on dark-adapted rats. Three days after eyedrop application, there was no difference in light responses between RCS rats treated with PSVue-550 eyedrops and littermates treated with Hanks buffered saline solution (HBSS) control eyedrops (Supplemental Fig. S3). Thus, PSVue-550 eyedrops do not directly affect functionality of retinal neurons or the course of ongoing retinal degeneration.

### *In vivo* detection of apoptotic RCS photoreceptors by whole animal imaging

**Figure 2:**
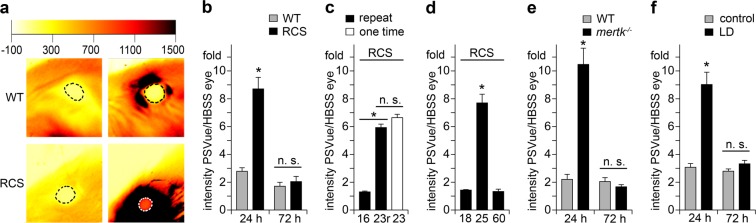
Live imaging of apoptotic photoreceptors *in vivo* by whole animal scanning. (**a**) Representative whole animal scans of p25 RCS and WT rats 24 hours after PSVue-550 or HBSS buffer eyedrop application as indicated. Intensity range on top shows false color scale. Encircled regions show quantified areas. (**b**) Quantification of fluorescence intensity as in (**a)** of p25 rats 24 and 72 hours after PSVue-550 application; n = 7 animals per group. (**c**) Quantification of fluorescence intensity 24 hours after eyedrops of RCS rats treated with PSVue-550 or HBSS eyedrops at p16 (16) with repeat at p23 (23r, black bar) side by side with p23 siblings that had not been treated before (23, white bar); n = 6 animals per group. (**d**) Quantification of fluorescence intensity 24 hours after eyedrops of RCS rats at p18, p25, and p60 as indicated; n = 5 animals per group. (**e**) Quantification of fluorescence intensity 24 and 72 hours after PSVue-550 application in pigmented *mertk*^−/−^ mice; n = 9 animals per group. (**f**) Quantification of fluorescence intensity 24 and 72 hours after PSVue-550 application in LD rats; n = 7 animals per group. (**b**–**f**) All bars show mean ± SEM. All asterisks indicate *P* < 0.05 by ANOVA and Tukey post-hoc test. n.s. indicates difference not significant.

Next, we imaged probe fluorescence in eyes of live, anesthetized WT and RCS rats after application of PSVue-550 to one eye and HBSS control eyedrop to the other (Fig. 2a). Fluorescence of contralateral eyes was measured to yield background fluorescence intensity, and PSVue-550-derived signals were quantified as fold increase over background specific to each animal. Using a whole animal scanner, recording fluorescence of the entire eye 24 hours after PSVue-550 application we found that fluorescence of RCS PSVue-550-treated eyes was elevated 8.7-fold (*P* < 0.001), while fluorescence of WT PSVue-550-treated and contralateral eyes did not differ significantly (*P* = 0.72) (Fig. 2a,b). Similar experiments using unconjugated fluorophore without the Zn-DPA targeting moiety did not increase the fluorescence signal (data not shown). Fluorescence of PSVue-550-treated RCS eyes decreased to control levels between 24 and 72 hours such that there was no difference in fluorescence between RCS and WT eyes or between treatments 72 hours after PSVue-550 application (*P* = 0.98) (Fig. 2b). Thus, PSVue-550 eyedrops transiently label degenerating RCS photoreceptors in the living eye.

We next sought to take advantage of the transitory nature of PSVue-550 labeling to repeatedly test the same animal, which will have utility in longitudinal studies. To this end, we applied PSVue-550 eyedrops to p16 RCS rats followed by whole animal live imaging 24 hours later. One week after the first eyedrop application, the same rats at p23 again received PSVue-550 eyedrops followed by imaging the next day. As controls, littermate p23 RCS rats not manipulated previously were also tested. These experiments showed that PSVue-550 eyedrops yield negligible labeling in RCS rats at p16, an age prior to onset of apoptosis (Fig. 2c, black bar 16). Repeating the non-invasive PSVue-550 labeling at p23 on the same rats yielded ~4.5-fold higher PSVue-550 signals compared to the signal detected at p16 (*P* < 0.0001) (Fig. 2c, black bar 23r). Notably, PSVue-550 fluorescence in littermate RCS rats that were tested at p23 alone did not differ from signals in p23 RCS rats that were tested twice (*P* = 0.092) (Fig. 2c, compare black bar 23r and white bar 23). Thus, PSVue-550 eyedrop labeling and live imaging allows longitudinal testing of the same animals on a weekly basis.

To further confirm that PSVue-550 labels only RCS photoreceptors that are in the process of undergoing apoptosis, we next tested RCS rats at different stages of disease. We confirmed normal morphology of RCS rat retina at p18 and complete loss of photoreceptors by p60 (Supplementary Fig. S4a–c). Caspase-3 immunoblotting further showed cleaved, active caspase-3 indicative of ongoing apoptosis at negligible levels at p18 and p60 as compared to robust levels at p25 (Supplementary Fig. S4d). Direct comparison of RCS rats at p18, p25, and p60 in PSVue-550 non-invasive imaging showed elevated signal as before at p25 while p18 and p60 signals were not significantly elevated and not significantly different from each other (*P* = 0.951). These results show that our method labels RCS rat retina only at an age with active apoptosis but not before onset of apoptosis or after photoreceptors are lost.

### *In vivo* detection of apoptotic photoreceptors in pigmented *mertk*^−/−^ mice

Like RCS rats, *mertk*^−/−^ mice lack expression of the clearance receptor MerTK^14^. Here, we studied pigmented *mertk*^−/−^ mice that had been backcrossed extensively to 129SvEms/J WT mice. As genetic background affects the retinal phenotype of *mertk*^−/−^ mice^15^, we first determined the time course of retinal degeneration in this strain (Supplementary Fig. S5). p28 *mertk*^−/−^ mice retained mostly normal retinal morphology and were thus chosen for this study. Live whole eye imaging performed 24 hours after PSVue-550 eyedrop application measured a 11.2-fold increase in fluorescence in PSVue-550-treated *mertk*^−/−^ eyes compared to contralateral eyes (*P* = 0.0038), while no probe-specific increase was detected in strain- and age-matched WT eyes (*P* = 0.87) (Fig. 2e). Like in RCS retina, PSVue-550 signals in *mertk*^−/−^ retina decreased to background levels by 72 hours after eyedrop application (Fig. 2e). These results show that PSVue-550 eyedrops allow live imaging of photoreceptor degeneration in rats and in mice, and, importantly, that pigmentation of eye tissues does not preclude detection of PSVue-550 signals emitted from the outer retina.

### *In vivo* detection of apoptotic photoreceptors in WT rats following light damage

In both RCS rats and *mertk*^−/−^ mice, debris of degenerating photoreceptors accumulates in the subretinal space due to defective RPE clearance phagocytosis forming a debris zone^14,16^. Such debris may expose PS and/or bind PSVue-550 yielding a larger PS signal from PSVue-550 eyedrops than in other forms of retinal degeneration where photoreceptor apoptosis is accompanied by clearance of dead or dying photoreceptors or their debris. We therefore tested detection of dying photoreceptors following light injury of WT rats whose RPE has intact clearance activity. Bright white light exposure triggered acute retinal degeneration as expected with early stage of retinal degeneration five days after light damage^2,17^ (Supplementary Fig. S6). PSVue-550 eyedrops were thus administered to control and light-damaged (LD) rats four days after light exposure followed by live imaging 24 hours later. Live whole animal scanning detected a 9-fold increase in PSVue-550 fluorescence in eyes of LD rats compared to contralateral eyes (*P* < 0.001), while no PSVue-specific increase was detected in control animals (*P* = 0.95) (Fig. 2f). As in the MerTK-deficient models, fluorescence intensity decreased to background levels by 72 hours after PSVue-550 application (Fig. 2f). These results demonstrate that PS exposure by photoreceptors is detectable using non-invasive live PSVue-550 imaging in early ongoing retinal degeneration induced acutely in LD WT rats with normal RPE debris clearance activity.

### *In vivo* detection of apoptotic RCS rat photoreceptors by retinal imaging

**Figure 3:**
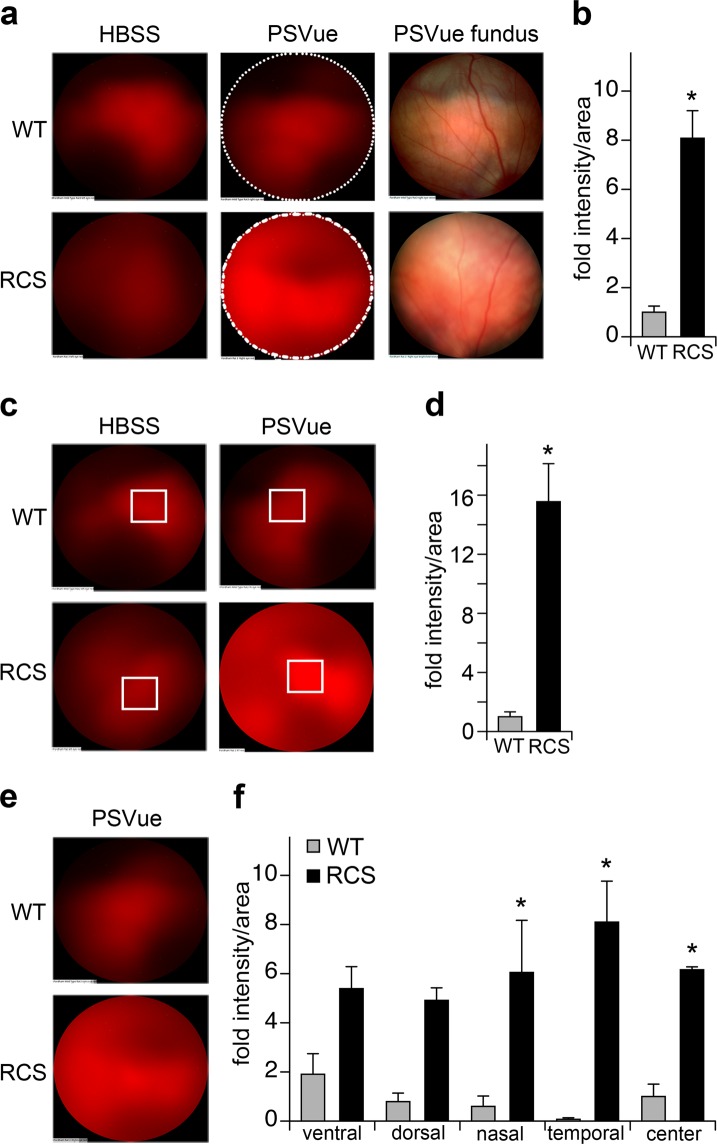
Live imaging of apoptotic photoreceptors *in vivo* by retinal imaging. (**a**) Representative fluorescence images of photoreceptors and fundus images of p25 RCS and WT rats 24 hours after PSVue-550 or HBSS solvent eyedrop application as indicated. (**b**) Quantification of fluorescence intensity after background subtraction in areas indicated by dashed lines. *P < 0.05 by Student’s *t*-test. (**c**) Photoreceptor fluorescence and (**d**) quantification as in **b** of fluorescence intensity maxima. *P < 0.05 Student’s *t*-test. (**e**) Photoreceptor fluorescence and (**f**) quantification of areas of fluorescent intensities specific to retinal quadrants. *P < 0.05; two-way ANOVA and Tukey post-hoc test. (**b**,**d**,**f**) All bars show mean ± SEM, n = 3 animals per group.

Small animal fluorescence imaging instruments are widely available to the scientific community and do not require expertise in vision science. However, specialized retinal imaging systems allow focusing on specific eye tissues. We therefore next tested PSVue-550 eyedrop treatment of p25 RCS and WT rats followed by fluorescence detection using a retinal imaging system. Fluorescence imaging at the site of degenerating photoreceptors detected an 8.6-fold increase in PSVue-550-treated RCS eyes over contralateral eyes (*P* = 0.0035) while WT eyes did not differ significantly (*P* = 0.29) (Fig. 3a,b). Fundus images were also recorded that confirmed known changes in retinal blood vessels in degenerating RCS retina^18^ (Fig. 3a). Retinal imaging further allowed quantification of separate retinal regions, which is meaningful as many forms of retinal degeneration progress with characteristic topography. In RCS eyes, the maximal PSVue-550-specific fluorescence was 15.4-fold as intense as the maximal non-specific fluorescence in the contralateral eye (*P* = 0.004) (Fig. 3c,d). Compared to PSVue-550-treated WT eyes, PSVue-550-treated RCS eyes showed increased fluorescence in all quadrants, but changes in the temporal quadrant were most pronounced and only nasal, temporal, and central regions were statistically different from WT (Fig. 3e,f) (inferior (ventral) *P* = 0.318, superior (dorsal) *P* = 0.149, nasal *P* = 0.022, temporal *P* = 0.004, center *P* = 0.003). These findings were in agreement with histology studies showing that RCS photoreceptor death progresses from the center to the periphery^11^.

## Discussion

The results shown in this study establish non-invasive *in vivo* detection in retinal photoreceptor neurons of PS exposure, the cardinal feature of early apoptosis. Our protocol requires only a one-time application of the non-toxic, commercially available PSVue-550 probe as an eyedrop. We have shown that PS signal detection by live imaging succeeds in three well established rodent animal models of retinal degeneration testing mice and rats, pigmented and albino animals, and inherited and induced retinal degenerative models.

We found that the small chemical compound PSVue-550 but not the annexin protein based pSIVA labels dying photoreceptors following application as eyedrop. We did not additionally test annexin V, the most widely used probe for PS exposure but it is unlikely to be useful as eyedrop given its structural similarity to pSIVA^12^. All three PS probes label dying photoreceptors in the models we explored here when applied *ex situ*. Here, we show that pSIVA and PSVue-550 yield similar staining of apoptotic outer retina in RCS rats following intravitreal injection and co-stain apoptotic RCS photoreceptors *ex situ*. We therefore conclude that lack of labeling by pSIVA following eyedrop application is due to its failure to reach the photoreceptor layer of the retina possibly due to its larger molecular size than PSVue^19^.

To generate the data set presented, we chose to use PSVue-550 as it matched well the excitation/emission settings of our whole animal fluorescence scanner. Our live imaging experiments consistently showed similar levels of background fluorescence in eyes not receiving PSVue-550 with and without retinal degeneration independently of the animal model tested. In non-degenerating retina, PSVue-550 eyedrops elevated fluorescence slightly but these increases did not reach statistical significance in any of the models tested. This was not due to lack of PSVue-550 penetration into ocular tissues in non-degenerating retina, which we directly tested to be equal in WT and RCS rats. Altogether, our results imply little interference from naturally fluorescent molecules in the eye/retina at the excitation/emission setting used to image PSVue-550 well justifying its use. However, PSVue is commercially available coupled to a variety of fluorophores spanning the visible light spectrum. We expect that such other PSVue probes applied as eyedrops will in principle yield similar labeling of dying retinal photoreceptors. However, whether detecting their specific fluorescence is possible with the low background and noise we found for PSVue-550 will need to be established.

We chose to perform *in vivo* imaging of PSVue-550 24 hours after eyedrop application. Our detection of the dye in the posterior eye by 3 hours after eyedrop application indicates that imaging for detection may succeed at earlier time points. However, at least for longitudinal studies 24-hour intervals in between anesthesia of individual animals may be advisable. By 72 hours after PSVue-550 eyedrop application, specific PSVue-550 fluorescence was no longer detectable in all three animal models indicating that PSVue-550 is completely cleared from the outer retina between 24 and 72 hours after application although retinal degeneration continues to progress. These findings are consistent with those from PSVue-550 imaging in rodent models of myopathy and ischemia-reperfusion where PSVue-550 was administered systemically^20,21^. We consider the rapid clearance rather advantageous as it minimizes the chance that the probe alters and possibly worsen the course of retinal degeneration. In support, we found no effect of PSVue-550 eyedrops on retinal function as tested by ERG 72 hours after application. Moreover, the loss of applied PSVue-550 within 72 hours allowed re-application and repeat measurements of the same animal during progressive forms of retinal degeneration. Importantly, we established specifically that re-testing the same animal one week after the initial testing was successful and that prior testing did not affect results of the repeat testing. Altogether our data demonstrate that PSVue-550 eyedrops will have utility for longitudinal studies.

In this work we focused on establishing non-invasive detection methodology for three different animal models that share a pan-retinal degeneration of photoreceptor neurons. However, given penetration of eyedrop-administered PSVue-550 irrespective of retinal degeneration, we expect that this method may be adapted to non-invasive detection of apoptosis by other retinal neurons as well. It is promising in that respect that principal PSVue probe detection of early apoptotic retinal ganglion cells following intravitreal PSVue-480 injection has already been successful in dissected, fixed tissue in a rat model of induced excitotoxicity^8^. In addition, retinal imaging may succeed in detecting focal regions of cell death for instance following acute laser injury although there will surely be a minimum size of damaged retinal region and extent of photoreceptor apoptosis for detectability. Expanding PSVue-550 eyedrop application and non-invasive imaging to these different disease models will thus require further studies.

PS exposure is a universal characteristic of early apoptotic cells and apoptosis is common to many if not most retinal diseases involving death of photoreceptors. Our results show that PSVue-550 imaging detects photoreceptor death in small rodent animal models. Such small animal models may be raised in sufficient numbers to afford testing multiple cohorts in analyses requiring animal sacrifice. However, besides the inherent value of reducing the overall numbers of animals needed for research non-invasive survival PSVue-550 imaging of retinal cell death will additionally be of significant scientific value. Longitudinal studies will be able to identify periods of cell death during development of retinal degeneration thereby allowing selection of specific informative animal ages for the in-depth histology studies. As PS exposure precedes irreversible changes during cell death^3,22^, anti-death therapies may be tested at the time of PSVue-550 fluorescence detection for efficacy in delaying retinal degeneration. Preselecting animals for experimental therapies based on PSVue-550 imaging will likely lessen data variability resulting from the use of animals based on ages alone without accounting for animal to animal variability in age of onset or speed of progression of retinal degeneration. Finally, PSVue-550 imaging does not require specific eye research expertise or equipment. The presence of either a retinal camera or a small animal imager is common to academic and industry research labs. PSVue-550 imaging offers a quick and economical approach to pre-screening any of the vast numbers of rat and mouse animal models that continue to be generated for the non-eye research in interdisciplinary collaborations.

Our study demonstrates no differences between mice and rats with respect to PSVue-550 detection of apoptotic retinal photoreceptors. Future studies may pursue utility of PSVue imaging in larger animals and, if successful, possibly translation to human studies where repetitive and completely non-invasive monitoring of apoptotic cell death in the retina will be of enormous value. Indeed, clinical testing of PS exposure by retinal ganglion cells is currently underway for glaucoma patients with annexin protein-based probes administered either through intravitreal injection^8^ or more recently intravenously^23^.

In conclusion, our results establish PSVue-550 eyedrop application and imaging as safe, completely non-invasive approach to monitoring photoreceptor death in small animal models of retinal degeneration. Given its simple protocol and the transient nature of PSVue-550 labeling allowing repeat testing we propose that this method will be widely applicable and useful for the field.

## Materials and Methods

Reagents were purchased from Thermofisher (Carlsbad, CA) or Millipore-Sigma (St. Louis, MO) unless otherwise indicated.

### Animals

Animals of both sexes were used. Pink-eyed dystrophic RCS rats (rdy/rdy-p) and Sprague Dawley (SD) wild-type (WT) albino rats were bred and raised to yield litters at defined age for experiments. *mertk*^−/−^ mice in 129 Sv background were raised by crossing B6-129-Mertk^tm1Grl^/J (Jackson Laboratories, strain #11122) for 9 generations with 129T2/SvEms/J (Jackson Laboratories strain #2065) WT mice. *mertk*^−/−^ mice were genotyped using published protocols and were found not to carry the rd8 mutation^24^. WT 129T2/SvEms/J mice were raised as controls.

Animals were housed under cyclic 12 h:12 h light-dark conditions with food and water ad libitum. Animals were housed in metal racks in non-transparent cages with metal lid supporting both water bottle and food pellets. This configuration provided sufficient shielding to minimize illumination inside cages. Light intensity varied from 10 lux in the back of the cage to 60 lux in front.

Anesthesia was induced by intraperitoneal injection of a mix of 100 mg/kg ketamine and 10 mg/kg xylazine.

### PSVue-550 dye preparation and fluorescent probe eyedrop application

Bis (zinc-dipicolylamine-550, (PSVue-550) was obtained from Molecular Targeting Technologies Inc. (West Chester, PA) and reconstituted according to the manufacturer’s instructions. The resulting 1 mM solution of PSVue-550 was stored in the dark at 4 °C and used within 14 days directly as eyedrop. 6–7 hours after light onset, anesthetized animals received in both eyes one drop of 0.5% proparacaine hydrochloride (Akorn, Lake Forest, IL) for 2 min as local anesthetic followed by one drop of 2.5% phenylephrine (Akorn) for 2 min to yield eye protrusion. This was followed by one 15 μl drop of 1 mM PSVue-550 solution or of pSIVA solution as provided by the manufacturer (Novus Biologicals, Littleton, CO) in HBSS while the contralateral eye received one eyedrop HBSS as control for 15 min. The eyedrop volume was chosen such that the eye cavity was completely filled without spillage outside the eye, and it may need to be modified depending on eye size. PSVue-550 at 0.5 mM was also tested initially on p25 RCS and WT rats with similar results. We used the 1 mM PSVue concentration for all studies such that probe availability would not be limiting for labeling. Rats were kept in darkness overnight before sacrifice and tissue harvest after 24 hours from the application.

### PSVue-550 dye penetration testing

P25 RCS and control WT rats received PSVue-550 or HBSS control vehicle as eyedrops under anesthesia and as described above. Animals were kept in the dark for 3 hours before sacrifice. The eyeball was rinsed with 10 μl HBSS to capture any remaining external probe, this rinse was analyzed as external sample. Following lens removal 10 μl HBSS were pipetted into the posterior eyeball, the re-collected liquid was analyzed as interior sample. Cleared samples were analyzed by dot blotting on nitrocellulose membrane followed by quantification of probe fluorescence intensity with a Sapphire Biomolecular Imager (Azure Biosystems, Dublin, CA) and direct comparison against a serial dilution of 1 mM PSVue-550 stock applied to the same membrane.

### Intravitreal injections

p24 WT and RCS rats were anesthetized. Using a dissecting microscope, 4 μl of 1 mM PSVue-550 or of pSIVA as provided by the manufacturer were injected into the right eye vitreous via the transscleral route using a SilFlex tubing and holder driven by a 10-μL glass NanoFil^TM^ microsyringe (World Precision Instruments, Sarasota, FL). An identical volume of HBSS vehicle was injected into the left eyes of the same animals. Animals were maintained in the dark until sacrifice and tissue harvest.

### Tissue dissection, live imaging, and fixed tissue histology

For tissue harvest, animals were sacrificed by CO_2_ asphyxiation followed by immediate eye enucleation and dissection.

For live imaging, neural retina and posterior eyecup tissue containing the RPE were dissected and separately mounted live in HBSS for immediate imaging on a TSP5 laser scanning confocal microscopy system (Leica, Mannheim, Germany). *For ex vivo* labeling, freshly dissected retinas from untreated rats were mounted in HBSS containing 2 μM PSVue-550 and 1/50 pSIVA followed by immediate live imaging. Single dye-labeled samples were tested as controls for channel-to-channel bleed-through.

In each assay, all tissues were imaged using identical settings and compiled and processed identically using Photoshop CS4.

For fixed tissue analyses, enucleated eyes were immersion-fixed in Davidson’s fixative followed by paraffin embedding and microtome sectioning, rhodopsin and nuclei counterstain labeling of sections as described in detail elsewhere^25^. For morphology analyses, tissues were stained with hematoxillin/eosin (H/E) according to standard procedures. Image stacks representing equal thickness were acquired using equal settings and collapsed to yield maximal projections of center and peripheral retina. Images were recompiled using Photoshop CS4.

### Whole small animal fluorescence scanning

Animals were anesthetized, and eyes treated with one drop of 2.5% phenylephrine for 2 min followed by one drop of 1% tropicamide (Akorn) for 2 min for pupil dilation. Animals were placed inside a Kodak FX-Pro imager (Bruker Bioscience Corporation, Billerica, MA) with one eye imaged at a time. Animals were imaged using 550 nm light excitation and 600 nm emission. Image analysis was performed with Multispectral FX-Pro software (Bruker). Fluorescence intensities in selected regions of interest (ROI) corresponding to each eye were quantified and the resulting values were compiled as fold intensity PSVue-550-treated eye over contralateral control eye that received only HBSS as eyedrop.

### Retinal imaging

24 hours after receiving PSVue-550 eyedrops or control solvent eyedrops on one eye and 4 to 6 hours after light onset rats were anesthetized. Age-matched RCS and WT cohorts comprising 3 rats each were tested side-by-side. A Micron-IV retinal camera was used (Phoenix Technology Group, Pleasanton, CA). Eyes to be imaged were covered with artificial tears and GONAK (both Akorn) before adjusting the position of the rat’s eye such that the camera’s eyepiece touched the cornea. 550 nm or white light illumination were used to acquire fluorescent and bright field fundus images, respectively. Images were acquired at different focal planes starting from the cornea to the back of the eye corresponding to the photoreceptor-RPE interface. Fluorescence intensities were quantified in selected ROI using Image J (National Institutes of Health, Bethesda, MD). Intensity values from contralateral untreated eyes were used to calculate fold intensity change in the PSVue-550 treated eye. For retinal quadrant analyses, data were normalized to the average of the center area of control samples.

### Electroretinography

Animals were dark-adapted overnight before recording scotopic responses under anesthesia and under dim red light exactly as described previously using a UTAS-E2000 visual electrodiagnostic system (LKC Technologies, Gaithersburg, MD)^26^. Stimuli were presented in order of increasing intensity as a series of white flashes of 1.5 cd-s/m^2^ attenuated to yield intensities from −1.8 to 0.2 log cd-s/m^2^. For each flash intensity, three to six recordings were averaged. For all recordings, a-wave amplitudes were measured from the baseline to the trough of the a-wave, and b-wave amplitudes were measured from the trough of the a-wave to the peak of the b-wave.

### Light damage (LD) induction

Light damage was induced adapting the protocol of Grimm and Reme (2013)^27^. p21 SD WT rats were maintained in the dark for 65 hours starting 5 hours after light onset before anesthesia and exposure for 1 hour to 10,000 lux of cool-white fluorescent light (Snap-on, 25 W LED Work-Light, broad spectrum light 380–760 nm). This was followed by 23 hours of exposure of the cage to 6000 lux using the same light source at greater distance with animals having access to food and water ad libitum and moving freely. Air temperature in the cage was monitored with an infrared thermometer (Tempgun TG1, NY) and maintained below 23 °C for the entire light exposure period by placing the cage into an open hood with ventilation. Following light exposure, animals were maintained in the dark before experiments. Age-matched control rats were maintained in the same environment and dark adaptation conditions except in normal room light during the 24 hours of bright light exposure.

### Sample lysis and immunoblotting

Whole eyes without the lens were lysed in 50 mM Hepes, pH 7, 150 mM NaCl, 10% glycerol and 1% Triton X100 freshly supplemented with protease inhibitor cocktail. Cleared lysates were boiled with reducing SDS sample buffer before separation on SDS-polyacrylamide gels and nitrocellulose membrane transfer using standard protocols. Membranes were sequentially incubated with primary and appropriate horseradish peroxidase-conjugated secondary antibodies and chemiluminescence reagent (Kindle Biosciences) followed by digital imaging using a Kwikquant Imager (Kindle Biosciences). Primary antibodies used were: caspase-3 (#9662, Cell Signaling, Danvers, MA), α-tubulin (#2125, Cell Signaling), and PSD95 (#MAB1598, Millipore-Sigma).

### Statistical analyses

Statistical analysis was performed by unpaired Student’s *t*-test using Microsoft Excel to compare any two samples or by one or two-way ANOVA followed by Tukey’s post-hoc test using Prism Graphpad 7.0 for comparison of multiple samples (LaJolla, CA). *P* values < 0.05 were considered a statistically significant difference.

### Ethical approval and informed consent

All experimental procedures were reviewed and approved by the Fordham University Institutional Animal Care Committee and complied with the policies and regulations regarding animal experimentation. They were conducted in accordance with the National Institutes of Health Guide for the Care and Use of Laboratory Animals (8^th^ ed.) and the ARVO Resolution for the Use of Animals in Ophthalmic and Vision Research.

## Supplementary information

Supplementary Info File #1
