# Supplementary material for: Non-invasive in vivo fluorescence imaging of apoptotic retinal photoreceptors
Source: Sci Rep. 2019 Feb 7;9:1590. doi: 10.1038/s41598-018-38363-z (PMC6367443; doi:10.1038/s41598-018-38363-z)

SUPPLEMENTARY MATERIAL

Non-invasive *in vivo* fluorescence imaging of apoptotic retinal photoreceptors

Francesca Mazzoni[1], Claudia Müller[1], Jonathan DeAssis[1], Deborah Lew[1], W. Matthew Leevy[2] and Silvia C. Finnemann[1*]

[1] Department of Biological Sciences, Center for Cancer, Genetic Diseases and Gene Regulation, Fordham University, Bronx, New York, USA

[2] Department of Biological Sciences, 100 Galvin Life Science Center, University of Notre Dame, Notre Dame, IN 46556

*Correspondence should be addressed to S.C.F. (finnemann@fordham.edu)

Supplementary Figures and Legends

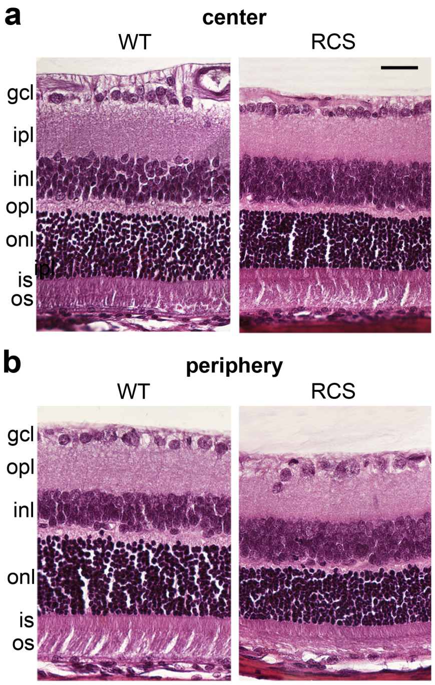

Supplementary Figure S1.

Largely intact RCS rat retina at p25 indicative of early stage retinal degeneration.

Representative light micrographs of central and peripheral areas from H&E-stained retina sections of p25 SD WT and RCS rats. Representative images of central (**a**) and peripheral (**b**) areas in WT and RCS retinal/RPE tissue, respectively. Note persistence but abnormal appearance of photoreceptor inner and outer segments in RCS eye. os, photoreceptor outer segments; is, photoreceptor inner segments; onl, outer nuclear layer; opl, outer plexiform layer; inl, inner nuclear layer; ipl, inner plexiform layer; gcl, ganglion cell layer. Scale bar, 50 µm.

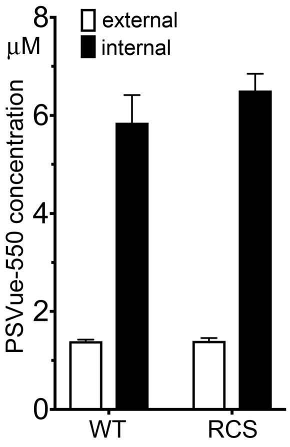

Supplementary Figure S2.

Penetration of PSVue-550 administered as eyedrop into the rat eye irrespective of retinal degeneration.

Quantification of PSVue-550 in HBSS buffer applied to the exterior of enucleated eyeballs (white bars) or of internal fluid retrieved from eyecups following extraction of the lens (black bars). Eyeballs were harvested 3 hours after PSVue-550 eyedrop application of p25 WT and RCS rats as indicated. Bars show mean ± SEM, n = 3 animals per group; PSVue-550 levels in the same compartment of WT and RCS rat eyes did not differ significantly as per two-way ANOVA.

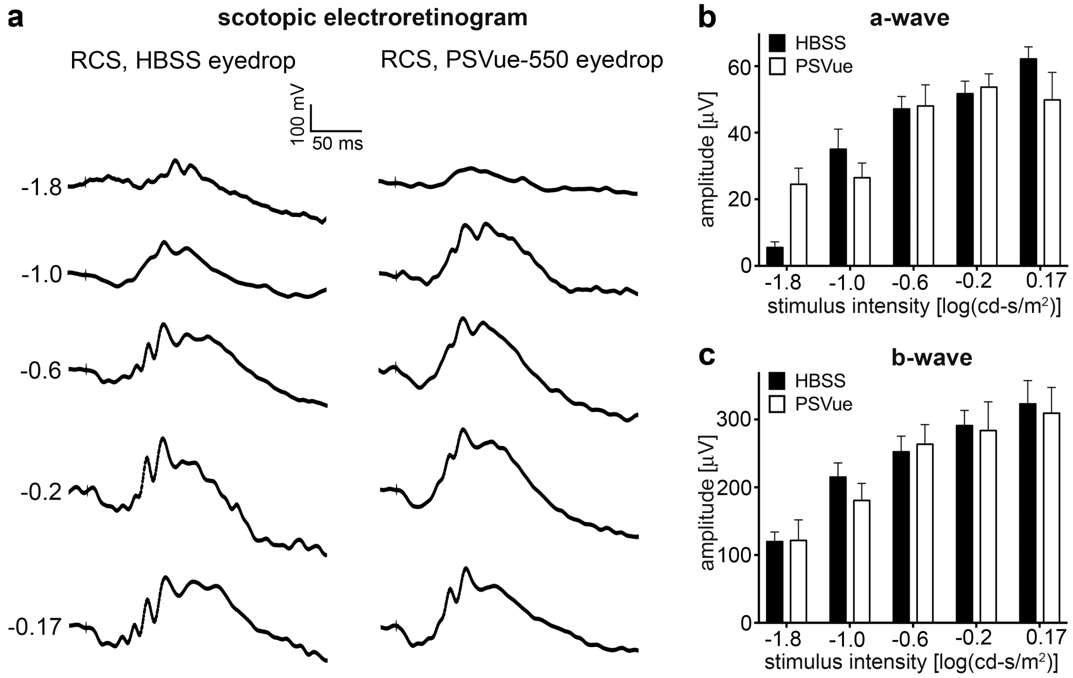

Supplementary Figure S3.

Lack of direct toxicity or adverse effects on vision of PSVue-550 eyedrops.

(**a**) Scotopic electroretinogram (ERG) recordings from littermate RCS rats 72 hours after PSVue-550 or HBSS eyedrops. ERG curves show averaged responses from one representative animal each. (**b**) a-wave and (**c**) b-wave amplitudes of ERGs as in **a**. White bars: PSVue-550 eyedrops; black bars: HBSS solvent eyedrops; mean ± SEM; *n* = 4 rats per group; differences not significant by 2-way ANOVA. Bars show one representative ERG experiment. The experiment was performed 4 times with identical results, each time testing 3 - 4 rats per group.

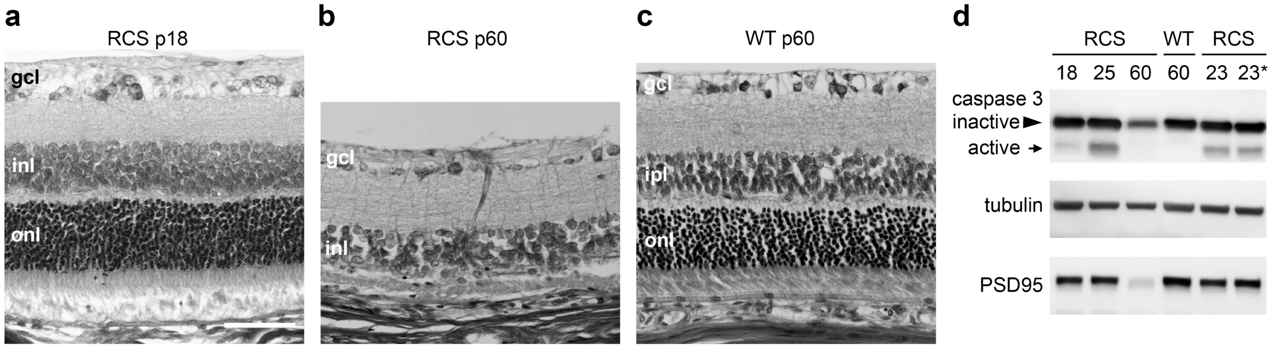

Supplementary Figure S4.

Characterization of RCS rat retina at ages prior to photoreceptor apoptosis (p18) and following complete photoreceptor loss (p60).

Representative light micrographs of central areas from H&E-stained retina sections of (**a**) p18, (**b**) p60 RCS rats and (**c**) p60 WT rats, as indicated. Note absence of outer nuclei layer of photoreceptor cell nuclei in p60 RCS rat retina. onl, outer nuclear layer; inl, inner nuclear layer; gcl, ganglion cell layer. Scale bar, 50 µm. (**d**) Representative immunoblot of whole eye lysates of RCS and WT rats sacrificed at ages as indicated by numbers above blots (p18, p25, p60, p23 and siblings p23* that had received eyedrops at P16). The same blot membrane was probed as indicated for caspase-3 whose cleavage is indicative of apoptosis, tubulin as universal cell marker, and PSD95 as synapse marker. Note increase of cleaved, active caspase-3 indicative of ongoing apoptosis between p23 and p25 but similar levels of PSD95 in RCS retina at p18 to p25 as in WT retina. Also note very little apoptosis in RCS retina at p18 and none at p60, at which age synapses are diminished. The experiment was repeated three times with identical results.

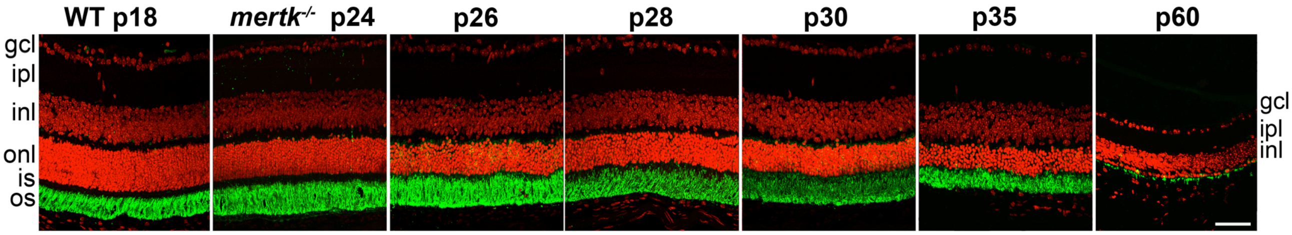

Supplementary Figure S5.

Early stage retinal degeneration in pigmented *mertk*$^{-/-}$ mouse retina at p28 and photoreceptor loss by p60.

Images show representative central areas of the retina of p18 WT and *mertk*$^{-/-}$ mice at ages as indicated. Nuclear staining is shown in red, rhodopsin staining indicative of photoreceptor rod outer segments is shown in green. p18 WT shows normal retinal morphology and tissue organization. Note outer nuclei layer diminishes by p60 indicating photoreceptor cell death. os, photoreceptor outer segments; is, photoreceptor inner segments; onl, outer nuclear layer; opl, outer plexiform layer; inl, inner nuclear layer; ipl, inner plexiform layer; gcl, ganglion cell layer. Scale bar, 50 µm.

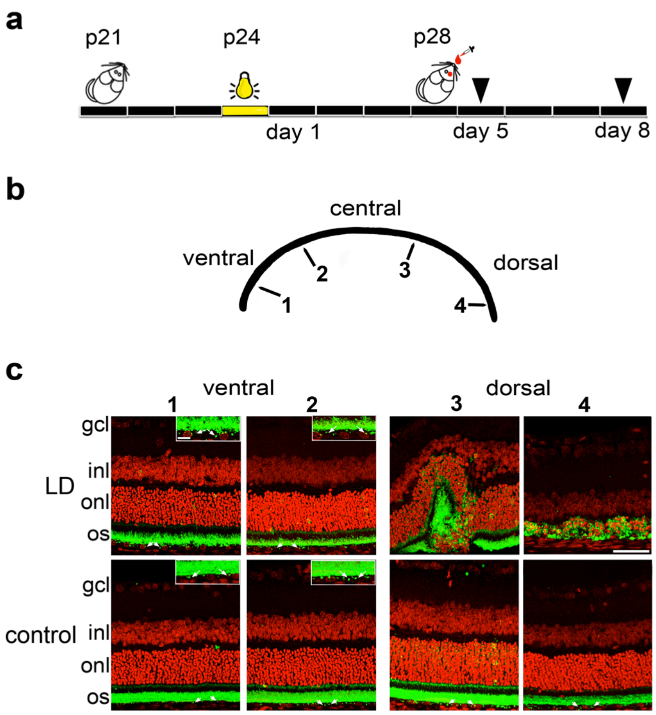

Supplementary Figure S6.

Induction of early stage retinal degeneration in p24 SD WT albino rats by light damage (LD).

(**a**) Experimental paradigm. Bar indicates animals were maintained in darkness for three days before bright light (LD) or room light (control) exposure at p24 for 24 hours. Rats were kept in the dark for the remainder of the experiment. Four days after LD, PSVue-550 eyedrops were applied. 24 hours and 72 hours later, animals were subjected to live imaging, indicated by arrowheads. (**b**) Map of retinal regions. (**c**) Representative images of ventral and dorsal regions (as in b) as indicated of retina from LD rats sacrificed 5 days after LD (upper panels) and from control rats (ctrl) (lower panels). Rhodopsin staining is shown in green and nuclei are shown in red. Scale bar, 50 µm. RPE, retinal pigment epithelium; ipl, inner plexiform layer; opl, outer plexiform layer; gcl, ganglion cell layer. Note thinning outer segment layer in ventral LD rat retina (1, 2) but severe inner and outer segment disruption and rhodopsin mis-localization to outer nuclear layer in dorsal regions (3, 4) as expected[17]. Insets show close-up of the RPE with opsin-positive phagosomes confirming that RPE in LD rats maintains clearance phagocytosis activity like control RPE. Scale bar inset, 10 µm.

Supplementary Information:

Uncut original immunoblots used to compile panels for Supplementary Figure 4d:

1st probe, entire membrane: Caspase-3

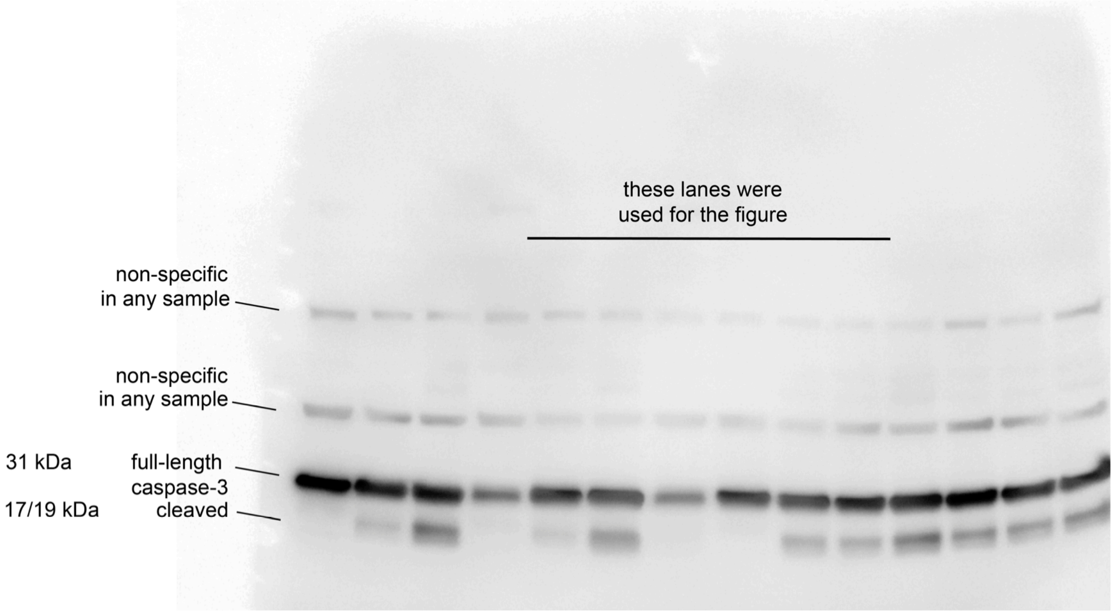

Membrane cut for 2nd probes: PSD95 and tubulin

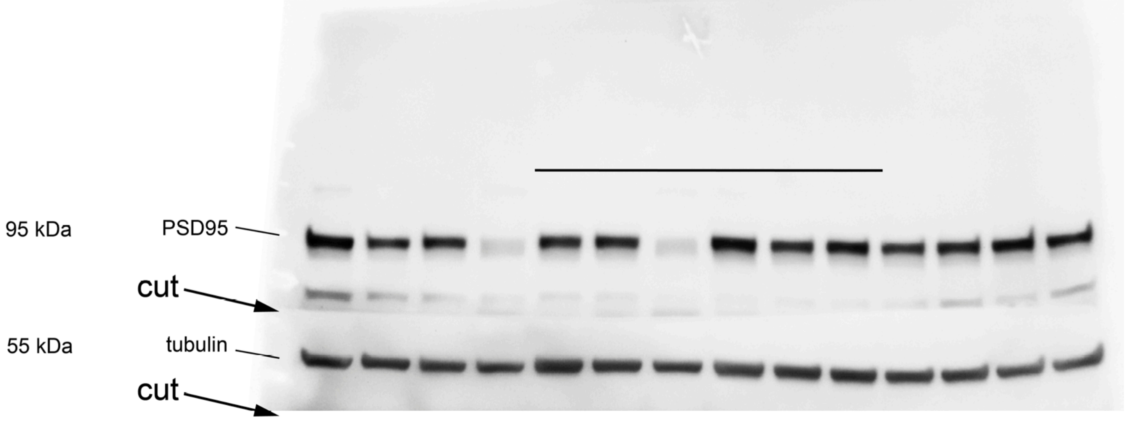

Supplement: Supplementary file 1 — Supplementary Info File #1 [file 41598_2018_38363_MOESM1_ESM.pdf]
